# Beaten but not down! Exploring resilience among female sex workers (FSWs) in Nairobi, Kenya

**DOI:** 10.1186/s12889-022-13387-3

**Published:** 2022-05-13

**Authors:** Rhoda Wanjiru, Emily Nyariki, Hellen Babu, Ibrahim Lwingi, Jennifer Liku, Zaina Jama, Mary Kung’u, Polly Ngurukiri, The Maisha Fiti Study Champions, Daisy Oside, Agnes Atieno, Agnes Watata, Demitila Gwala, Ruth Kamene, Mary Akinyi, Faith Njau, Chrispo Nyamweya, Pooja Shah, Monica Okumu, Helen Weiss, Rupert Kaul, Tara S. Beattie, Joshua Kimani, Janet Seeley

**Affiliations:** 1 Partners for Health and Development in Africa, Nairobi, Kenya; 2 London School of Hygiene and Tropical Medicine, London, UK; 3 Department of Medicine, University of Toronto, Toronto, Canada; 4 MRC International Statistics and Epidemiology Group, London, UK

**Keywords:** FSW, Sex work, Resilience, Stigma, Violence, Kenya (East Africa)

## Abstract

**Background:**

In Kenya sex work is illegal and those engaged in the trade are stigmatized and marginalized.

We explored how female sex workers in Nairobi, Kenya, utilize different resources to navigate the negative consequences of the work they do.

**Methods:**

Qualitative data were collected in October 2019 from 40 FSWs who were randomly sampled from 1003 women enrolled in the Maisha Fiti study, a 3-year longitudinal mixed-methods study exploring the relationship between HIV risk and violence and mental health. All interviews were audio-recorded, transcribed and translated. Data were thematically coded and analyzed using Nvivo 12.

**Results:**

Participants’ age range was 18–45 years. Before entry into sex work, all but one had at least one child. Providing for the children was expressed as the main reason the women joined sex work. All the women grew up in adverse circumstances such as poor financial backgrounds and some reported sexual and physical abuse as children. They also continued to experience adversity in their adulthood including intimate partner violence as well as violence at the workplace. All the participants were noted to have utilised the resources they have to build resilience and cope with these adversities while remaining hopeful for the future. Motherhood was mentioned by most as the reason they have remained resilient. Coming together in groups and engaging with HIV prevention and treatment services were noted as important factors too in building resilience.

**Conclusion:**

Despite the adverse experiences throughout the lives of FSWs, resilience was a key theme that emerged from this study. A holistic approach is needed in addressing the health needs of female sex workers. Encouraging FSWs to come together and advocating together for their needs is a key resource from which resilience and forbearance can grow. Upstream prevention through strengthening of education systems and supporting girls to stay in school and complete their secondary and/or tertiary education would help them gain training and skills, providing them with options for income generation during their adult lives.

## Introduction

In Kenya and many countries in sub-Saharan Africa, sex work remains illegal and those who make a living through this work are stigmatized and marginalized by others in the community [1]. Female sex workers (FSWs) have a higher risk of acquiring HIV and STIs compared to women in the general population [2, 3]. In Kenya, the HIV prevalence among FSWs is 29.3% compared to 6.6% among women in the general population [4]. As reported in other parts of the world, FSWs are also frequently victims of violations of human rights and basic human decencies such as having inadequate access to relevant health care services, a lack of justice due to punitive laws criminalizing sex work [5], and unlawful arrest and detention [6]. In Kenya, the penal code CAP 63 of the constitution criminalizes sex work [7]. This potentially creates the environment for law enforcers to arrest, harass, and assault sex workers without consequence, and makes it impossible for them to seek legal reprieve when abused by their clients or other people in the community, including their families and intimate partners [8]. Additionally, punitive laws can leave room for systemic discrimination to thrive in many institutions, including healthcare facilities [9]. Sex workers may face additional hostilities from their communities as it is believed they aid in the spread of HIV, and that they engage in ‘immoral’ activities [10]. The anticipated, enacted, and perceived stigma towards sex workers can be a driver of HIV acquisition as it often discourages access to HIV treatment and prevention services [11]. Internalized stigma - exemplified by reports of feeling ashamed and/or embarrassed by their work - is likely to create feelings of guilt, self-loathing, and low self-esteem [10].

Earning a living from sex work is both challenging and a taboo subject in many communities in Kenya. Hence, many FSWs experience overlapping and sequential adversities and disadvantages. These include housing insecurity, financial hardship, violence from different perpetrators including family members, partners, clients, police, and goons. They also face stigma and discrimination from service providers, family, people in their local community and others. The disadvantages that FSWs face, may interact throughout their lives creating a cycle of suffering and deprivation from which it is hard to escape [12].

### Theoretical framework

**Fig. 1:**
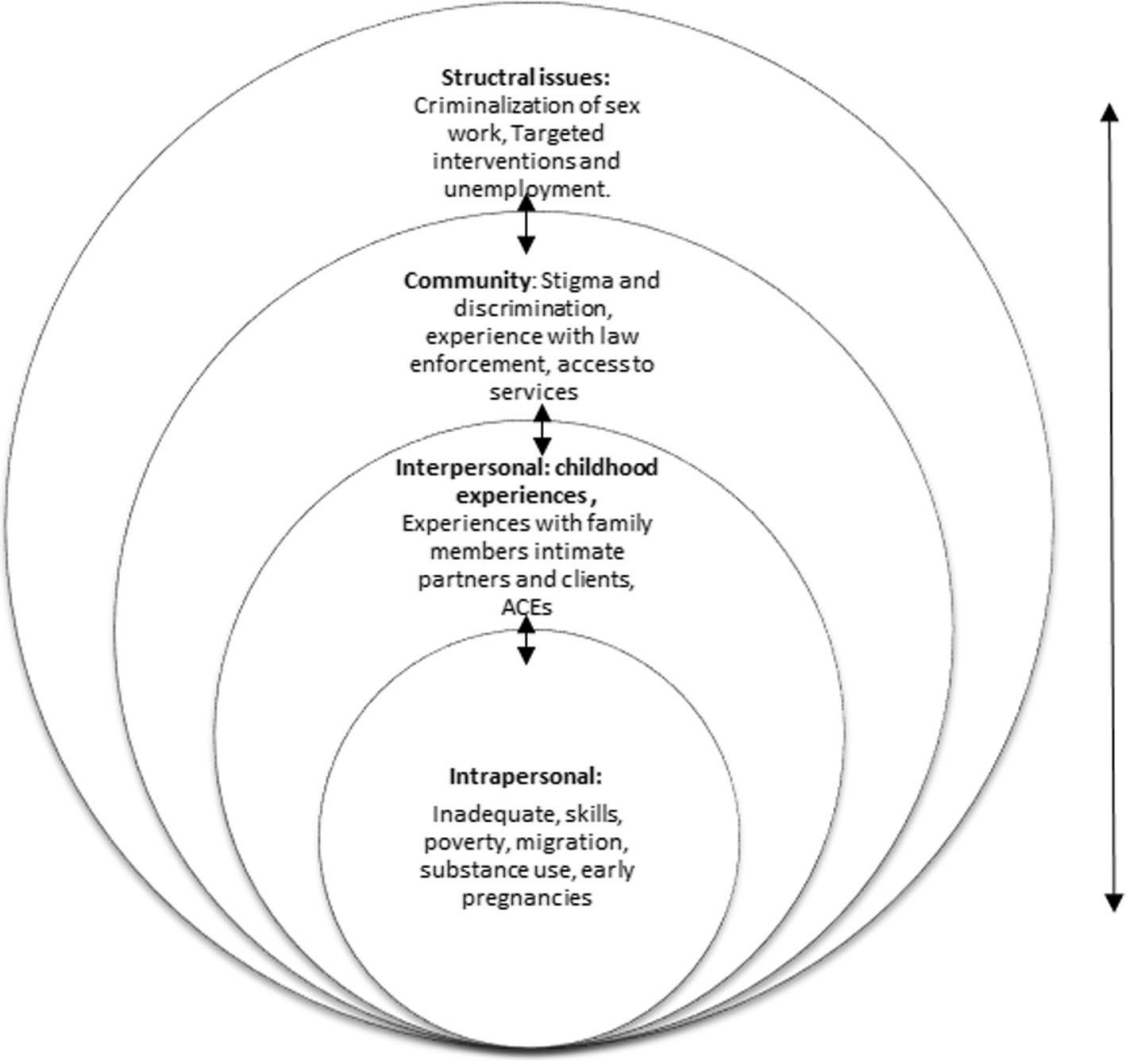
Bronfenbrenner’s ecological systems theory adapted for female sex workers [13]

In this paper, we explore how different experiences over the life-course of FSWs interact to keep them disenfranchised or marginalized, especially in Kenya and explore how they cope, survive, and even thrive, despite these circumstances. We will draw on Bronfenbrenner’s ecological systems theory [13] and resilience theory [14] for our analysis. These two theories are not FSW-specific. However, combining ecological systems theory with resilience allows us to consider the different societal levels at which FSWs experience marginalisation and demonstrate their resilience through the ways they manage the challenges they face. Bronfenbrenner’s ecological systems theory posits that intra-personal factors form the most proximate level and include poverty, early pregnancy, lack of education and skills, and harmful behaviours such as substance misuse [12, 15]. Inter-personal factors form the second level and include experiences with family members [16], Adverse Childhood Experiences (ACEs), intimate partner violence, and interaction with clients [17]. The third level is structural, this involves the interaction of sex workers with factors such as institutional stigma and discrimination [18], criminalization of sex work, and interaction with law enforcers [19], as well as targeted interventions provided by sex workers HIV treatment/prevention programs, gender-based sexual violence responses, and sex worker empowerment programs. Together, these multi-level factors may interact to determine the quality of the lives of sex workers. FSWs often experience high levels of adversity, including ACEs [20], which may be linked to negative outcomes later in life [21, 22]. Disadvantages during childhood may translate into mental health and substance use problems and other vulnerabilities in adult life [23]. In their adult life, sex workers often continue to suffer physical, sexual, emotional, and economic violence at home and at work due to factors such as stigma and discrimination, criminalization of sex work, and gendered power dynamics [1, 5, 9, 24, 25]. Together these factors may act synergistically in causing a disproportionate burden of HIV/STIs and poorer mental health outcomes among sex workers [2, 26] (Fig. 1).

We also draw on resilience theory [14] to explore why some FSWs can thrive despite experiencing adversities at the different levels laid out in the ecological systems theory. Research in Africa has explored some of these adversities and how sex workers respond to violence and other human rights abuses [27]. Resilience can be defined as either a process or an outcome depending on the context being described [28]. As a process, resilience has been described as being dynamic and combining both positive adaptations as well as adverse elements [29]. It is a product of risk exposure and promotive factors that may either reduce or overcome the undesirable effects of the lived experiences [15]. Promotive factors include both assets and resources. Assets are a person’s intrinsic factors such as life skills, faith, hope, possession of academic and competency skills, emotional stoicism, and charisma [14]. Resources can be conceptualized as extrinsic factors and include social support, community empowerment, and access to financial resources [30]. Resilient individuals are those that can adapt to extraordinary circumstances, achieving positive and unexpected results in the face of adversity [28]. In this paper, we will focus on resilience as a process to show how FSWs in Nairobi continuously face adversities throughout their lives and how they utilise the little opportunities they can access to make do and remain hopeful for the future.

## Methods

### Study setting and design

Nairobi is the capital city and the main economic centre of Kenya. There is a plethora of entertainment venues to cater to those with disposable incomes, and sex work thrives in these settings. Women engaged in sex work are active during the day and at night and congregate in specific places where they seek out clients. These places include particular streets, bars, lounges, restaurants, massage parlours, strip clubs and sex workers’ rooms in lodges. Once negotiations on the terms and payments for the sex are done with their clients, sex can happen in the same venues if the bars have lodgings, or other rented spaces as well as in cars or behind buildings. In 2017/2018 Nairobi County had the largest population of sex workers in the country, accounting for 25% of the estimated 206,000 women who sell sex on a peak day in the country [31].

Sex workers in Nairobi are encouraged to access much-needed HIV prevention and care services from seven standalone Sex Workers Outreach Program (SWOP) clinics, funded by the CDC-PEPFAR. These clinics are strategically situated across Nairobi County and serve as safe spaces for at least 33,000 FSWs. One clinic is located in downtown Nairobi while the others are within the informal settlements, to take the services closer to where the majority of sex workers live, and to cover the key sub-counties within Nairobi. The clinics provide accessible and friendly comprehensive HIV prevention and treatment services to key populations (FSWs and Men who have Sex with Men [MSM]) in the city [32].

Our findings come from interviews with FSWs residing in Nairobi, Kenya, collected as part of the Maisha Fiti study, a 3-year UK Government-funded mixed-methods longitudinal study which began in 2019 and enrolled a random sample of 1003 FSWs who access HIV prevention and treatment services from the SWOP clinics, in Nairobi County. The study aimed to explore risk factors for genital inflammation and antiretroviral (ARV) uptake and adherence, among women already enrolled in the HIV prevention and treatment services. From a sampling frame of all women aged 18–45 years (*n* = 10,292) who had attended a SWOP clinic in the 12 months preceding the study start, 1003 were selected. In-depth interviews (IDIs) were then conducted with a random sample of 40 of these 1003 participants. The interviews aimed to understand the women’s interpretation and experiences of violence, mental health, alcohol and substance use, and how these relate to HIV risk behaviours. The inclusion criteria for the study were participants who were assigned female sex at birth, self-identified as sex workers, accessed SWOP services within 12 months before contact with the study, did not have pre-existing conditions and were voluntarily willing to give written consent to participate in the study. This paper makes use of the qualitative in-depth interview data collected from these 40 women.

The qualitative study team comprised five social scientists, two of whom were interviewers. The interviewers had at least a degree in social sciences, updated ethics and human subject protection training and had undergone sensitization on working with key populations. All the study team members went through a detailed protocol training for the objectives of the study to be clear. The sensitization training is a mandatory continuous training within the research site that helps staff handle key population issues without stigma or imposing their beliefs and values on work issues. The interviewers had no prior relationship with the participants which may have minimised desirability bias. Following informed consent procedures, women were asked to provide detailed life stories; narrating specific events or aspects of their lives, including sex work initiation, sex work and violence experiences, and how these relate to mental health, alcohol and substance use, and Post Exposure Prophylaxis/ Pre-Exposure Prophylaxis/ Antiretrovirals (PEP/PrEP/ARV) uptake and adherence.

All interviews were conducted either in Kiswahili or English and lasted between one and one and a half hours, depending on the level of detail in which the participants gave their stories. The interviews were conducted in two rooms within the research clinic that were dedicated for this purpose and all necessary arrangements for confidentiality and support in the event of distress were put into consideration. Following each interview, the interviewers wrote a detailed script, which described the interview including nuances that would not have been captured on audiotape (such as a woman’s demeanour). Women were asked if they consented to the interview being audio-recorded. Thirty-eight of the 40 women gave their consent to be audio-recorded. The interview recordings were transcribed and then translated.

### Data analysis

The research team met after every two interviews had been conducted to discuss matters arising as data collection progressed, and to augment the interview guide as new themes or questions emerged. During these meetings, the interview notes from the social scientists were read and discussed to first identify, then refine and define the themes and sub-themes that were used to create a codebook. When all the interviews were completed, one interview script was randomly chosen and coded by four team members, who then met together and ran through the coded interview to check whether the codebook was understood in the same way by all members. This exercise confirmed that the four team members approached the exercise uniformly and the codebook was finalized. The 40 transcripts were then divided amongst the four team members who coded independently and analyzed the transcripts thematically, a process which was managed using Nvivo software.

### Ethical approvals and considerations

All the research activities were performed in accordance with the declaration of Helsinki**.** Ethical approval for this study was obtained from ethics boards at the Kenyatta National Hospital/the University of Nairobi (KNH/UoN), the London School of Hygiene and Tropical Medicine, UK and the University of Toronto, Canada. Research permission was granted by the National Commission for Science, Technology and Innovation, Kenya. The study participants were informed about the study through a detailed participant information sheet which was provided in written format and read aloud to all participants. They then provided their written informed consent in accordance with the ethics requirements. Audio-recorded data were uploaded to a secure server following each interview and deleted from the device. All data were stored using unique identifiers (IDs)-the study IDs were formatted as MF 0001- MF1003- in a password-protected server to maintain confidentiality. For this paper, we use pseudonyms to identify material drawn from different women’s interviews while protecting their identities. All participants were reimbursed for their transport to and from the study site. They were also treated for any presenting illness and referred to a psychologist on-site if they needed extra support. Those presenting with mental distress during and after the interviews were closely monitored by the study psychologist for support and onward referral where necessary.

## Results

At the time of the study, all the participants were residing in Nairobi and its environs and sold sex within the county but not in the localities where they lived. Their ages ranged from 18 to 45 years with a mean of 32 years. Most had some years of schooling but only two of the women had proceeded to tertiary level education. Before entry into sex work, 39 out of 40 women had had at least one child. Providing for the children was expressed as being the main reason the women joined sex work. All of the participants first sold sex at ages 18–24 years, with a mean of 21 years.

Through our analysis, and drawing on our theoretical framing, we found that the difficult circumstances experienced by the women in their lives could be categorized into three broad themes: facing adversity, building resilience and hopes for the future, which are elaborated further below. These broad themes are used to order our findings. We show how these themes relate to the layering provided by ecological systems theory in Table 1, below.

**Table 1:** A summary of the results by theme and mapped to the Ecological Systems Theory layers (shown in Fig. 1)

Themes	Facing Adversity	Building Resilience	Hopes for the future
Ecological systems theory			
Intrapersonal	-Mental Health problems which may have stemmed from the adverse experiences. The women reported inadequate support for mental health services.	-Adopting assorted coping mechanisms as described in the consequent ecological systems levels.	-Praying for better prospects in future kept most of the participants resilient.
Interpersonal	-Adverse childhood Experiences; one or a combination of: physical, sexual, and/or emotional abuse from an early age which was traumatic -Intimate partner violence	-Motherhood as a resource for resilience where the women reported working hard and holding on for their children to have a better life than they did. - Moving from abusive relationships as well as sourcing livelihoods for self and family most achieved this by joining sex work	Investing in their children’s future via saving groups and table banking. They hoped to get married to a good partner and have a good life
Community	-Sex work challenges;- The women reported that earning a living through sex work was painful and difficult. They not only faced violence from clients but also rough treatment during sex -Social harm and stigma where FSWs reported being viewed as immoral and homewreckers and deserving of the injustices they face.	-Engagement with HIV Prevention and Treatment services; women reported feeling empowered to safely engage in sex work after accessing the services and health education on safe sex. -Social capital; the sex workers reported benefiting from a support system with other sex workers that developed while using the safe spaces within the SWOP Clinics., Over time friendship grew amongst the clinics’ attendees leading to strategies being devised to encourage and help each other out.	Hoping their children will have better lives. Forming sex workers’ collectives to fight for their human rights and dignity
Structural	Criminalisation of those engaged in sex work. High unemployment rates .	Taking up alternative sources of income generation besides sex work	Exiting sex work and finding success in alternative means of livelihood. Decriminalisation of sex work

Below we present the findings on the three main themes, moving from adversity to the building of resilience and a more hopeful future.

### Facing adversity

All the women described pain and difficulties in their lives, either from childhood, in their married lives for those that were or reported ever being married, and/ or in their line of work, with most reporting on all three.

#### Adverse childhood experiences

Women reported growing up in poverty, and some were orphaned while still young, often resulting in them dropping out of school to fend for themselves and their siblings. Some participants described experiencing one or a combination of physical, sexual, and/or emotional abuse from their parents and guardians from an early age which was rough and traumatic. A toxic home environment coupled with inadequate resources for the participants to complete their education or to learn a skill played an important role in putting many women when they were adolescents in vulnerable positions.

*“The thing that was hurting me so much, there was this one man who was our neighbour. He was an old man and I used to be sent by my mum to take him food. That man used to hurt me because I did not know it was bad, I did not tell anybody. He would remove his thing and touch me with it.” (*Makena, 32 years old)

As a result of their different adverse experiences during childhood, many women became pregnant and married or lived with the fathers of their children while they were still teenagers or young adults.

#### Intimate partner violence

Life frequently transitioned from abuse in the homes they grew up in, to emotional, physical or sexual abuse by their intimate partner. Wawira (28 years old) recalled her experience:

*“Yes, it was beating every time. You have done wrong, or you have not done wrong you are beaten … And he will beat you that if you don’t escape you will die. That is why the day I left I said enough is enough. I had a small baby who was four months old … ”*

Another participant described having a partner who continuously sexually abused her, even immediately after childbirth. The sex felt obligatory to her because she was married to him.

Intimate partner violence within these early relationships when they were young, was the main reason many participants left the relationship in search of peace and safety for themselves and their children.

#### Sex work challenges

For most of the women we talked to, when these relationships broke down, returning to their parent’s home was not an option so they stayed with their friends, rented cheaper houses or sometimes slept on the streets with their children while thinking of the next steps in their lives. To care for their children, some took up jobs such as a waitress in bars, working as house helps or assisting in salons while others tried out businesses such as street vending, roadside food kiosks and selling illicit alcohol. However, these jobs paid poorly, and the income the women could make was not adequate for their needs. Participants mentioned turning to their women friends who seemed to be doing better in life for advice, who then introduced them to sex work.

Sex work was expressed as being a form of reprieve by most participants as they could provide for their children and sometimes share their earnings with extended families. It also offered some autonomy and independence. However, it was not easy money, as the participants felt earning a living through sex work was painful and difficult; they not only faced violence from clients but also rough treatment during sex. They had to show forbearance and resilience to survive and make the money they needed.

*“My money I get it painfully. You must endure because sometimes you get a person that is rough on you, he will sleep with you and you bleed. That money you see you don’t get it easily it is by pain.”* (Nakesa, 40 years old)

The women, as they recounted their experience, not only talked of physical violence but also emotional abuse through humiliation, and sexual abuse, especially a refusal to use condoms.

*“I have experienced violence maybe someone beats you even with a bottle, in front of your friends telling you to go with him, because you have refused. Because he wants you to do it without a condom, you refuse he beats you even with a metal rod, another one even wants to stab you with a knife. Those are the types of violence I have experienced*.” (Dorice 39 years old)

Women also said that they frequently faced harassment and abuse from police officers or knew another sex worker who had. The law enforcement officers often arrested sex workers, detained them without presenting them to court as required by the law, beat them up, and demanded sex and money from them in exchange for their release. These same policemen were expected to be the protectors of their rights and when they turned into abusers the sex workers were left with little recourse to seek justice for wrongs committed against them.

#### Mental health and social harm

Violence from different perpetrators was often reported as a cause of poor mental health by participants. They also felt that there were inadequate support systems for those who have been abused, such as counselling services and social support, which led to a further deterioration in their mental health and experiences of depression. The victims of violence were often forced to be resilient and learn to live with their experiences of abuse. This could in turn negatively affect their productivity and how they related with their children, family and friends.

*“My story is really bad. There is a time I contemplated suicide by jumping into a river to leave the problems behind. I left my husband’s house he would beat me so much there are days I spent the night in the shamba [garden], he would wake the kids and throw them out too. When I was in that marriage I had so many problems.* (Wawuda, 36 years old)

When the women could not cope with their everyday stressors, many mentioned using alcohol and/or drugs such as cannabis or khat to ease their pain. Suicidal thoughts were mentioned by several women.

*“At the very beginning, because I had hit the wall, I had bad thoughts. I had thought I will buy poison, kill all my children and kill myself. I did not have money and was looking for a little to buy rat poison and we all die at night. When I slept, I thought about why I want to kill these children. I woke up and cried so much, and realized that they are innocent.”* (Naserian, 29 years old)

However, women described being aware of how much can go wrong while intoxicated, including condomless sex as a result of diminished perceptions of risk and loss of income because they forgot to ask for money or negotiate for too little. The women were well aware that their problems persisted after the effects of the drugs subsided.

Sex workers reported being viewed as immoral and homewreckers by the wider community and hence were treated with disdain. They felt the wider community believed that they deserved the atrocities meted out on them such as violence because they ‘steal husbands’ and/or steal from their clients too. Hence, most participants hid what they did for a living from family, friends and their communities and this led some to feelings of self-loathing and embarrassment.

### Building resilience

The participants expressed different ways in which they coped and made do while faced with adversities. These were both individual and collective activities.

#### Sourcing livelihoods for self and family

Despite periods of hopelessness, especially before joining sex work, a majority of the study participants did not give up on life and eventually joined sex work for financial security. *“There was nothing else I could have done even if I didn’t like the idea. I was also not for the idea of having a husband. So, I started sex work and have been doing it since.”* (Teddy 34 years old). Teddy had a will to survive and provide basic needs and safety for her children. Nevertheless, sex work comes with its set of challenges such as violence, stigma, and social harm which make it not an ideal source of income as most participants reported. Despite this, most remain stoic and focused on providing for their families, determined to be resilient in the face of their problems.

#### Social capital

Most of the sex workers reported benefiting from a support system with other sex workers, their local community, that they had devised to encourage and help each other out. The support included but was not limited to, financial and emotional support, sharing of health education information, and linkage to health care services. For example, Almasi (28 years old) described the role of friends in her life: *“Your friend will come and tell you what happened, and we encourage each other to take action. Like in case of a condom burst you advise your friend to go for the 28 days drug and if she does not know where the hospital is you take her. You can also take her to SWOP where she can get help.”*

Given that the women reported having little familial, community, social or structural support, the friendships and grouping amongst their peers offered many women comfort and a means of thriving despite the many challenges. This social capital as a resource for resilience was cherished among sex workers.

Another resource for resilience was reported as being a safety net provided through their regular partners. Even though these are paying partners, participants said that these men would treat them with respect compared to casual clients. They mentioned few or no surprises while interacting with the regular partners as they know these men. Regular partners were also perceived as friends who provided a form of emotional support, as they could offer a shoulder to lean on even when they are not buying sex.

#### Engagement with services

Most of the participants said they had little or no knowledge of HIV prevention before engaging with the SWOP clinic services. However, women reported that after accessing the services and information from the SWOP `sex worker friendly’ clinics, they no longer accepted to have sex without a condom because they understood that the nature of the relationship with their clients is purely transactional. Some participants also reported using PrEP because they are aware of the risk they took, and should there be a condom burst, they were safe.

*“Ok, God has helped me. I never used to protect myself. I now don’t accept to sleep with anybody without protection because I know this one is not a husband, I am not looking for a man first thing, so without protection, there is no friendship there.”* (Felistas, 32 years old)

The women who took steps to cut down on excessive alcohol use or use of drugs like marijuana, mentioned their children as the main motivator. Some cited that when they were high, they would be rude and beat up their children. However, in the interest of being good examples and not stressing their children, they decided to reduce their reliance on alcohol and other substances:

*“I used to get so drunk that even when my children see me, they would start crying. Now I said surely this is losing my children I am misleading them. I had to tell even the people from SWOP and they told me to reduce. Because these children of yours first you will give them stress, second, they will join that behaviour you are showing them, third, the drugs (ARVs) will not work you will even forget to take”.* (Naserian, 34 years old)

Being mothers was also the biggest resource from which the women found the strength for building resilience. For example, for the few who reported suicidal thoughts, even when they felt at their lowest and every reason to live was gone, their children gave them a purpose to fight on.

### Hopes for the future

Despite all they had experienced, most of the participants had hopes for a better future where they were not sex workers. Indeed, only one of the forty women felt that sex work met her needs and she had no plans to leave. Many prayed to God to make a way to a brighter future, while some hoped to meet a rich man who would pull them from poverty. Some were actively making plans to transition to their envisioned future, through another job or a business. To see this through, some had joined savings groups and micro-enterprise ventures from which they could get dividends and get capital to start their businesses. Some other participants said that they wanted better lives for their children and were investing in their education in the hope that they become more successful than them. For example, Phiona (37 years old) put her hopes in her children: *“I sell sex and pay school fees for my children; I pray to God that he helps them to pass the level that I reached.”*

For most participants, there was an abiding hope in God to help them to a better future, and for God to give them the resilience to manage their present trials.

## Discussion

The environments in which most of the sex workers grew up, work and continue to live, were reflected in their life stories through which they described adversity and marginalization. However, resilience resonated throughout their life stories. The challenges the women described including difficult childhood experiences, intimate partner violence, work-related violence, harassment by authorities, and stigma and discrimination have been reported by sex workers from many other lower and middle-income country settings [33–35]. We found that ACEs such as sexual abuse, physical assault, emotional and physical neglect were a precursor for entry into sex work, in line with findings by Stoltz and Shannon in their study in Vancouver [36]. After starting sex work, female sex workers continue to experience challenges that negatively affected their mental health, self-worth, confidence and health. These challenges often led to harmful alcohol and substance use as a coping mechanism. The violation of human rights meted out against female sex workers in our study is supported by literature from other settings in sub-Saharan Africa and includes unlawful arrest and detention, extortion, and violence [27]. These experiences have been found to affect their mental, physical, and social well–being [27, 37]. Nevertheless, the participants in our study fostered resilience as they struggled to utilise the little they have as they hope and work for a better future. This fits in well with the ecological systems theory as their life is influenced at each of the levels of influence in their surroundings and circumstances.

The sex workers described how they have navigated and built resilience with the resources and avenues available to them: finding support and comfort in their immediate local community of peers. In different parts of Africa FSWs have been found to have various individual, formal, and informal tactics that they use in combination and/or separately to navigate sex work in environments of stigma, discrimination, and lawlessness [27]. We found that FSWs reported coming together to help each other financially, with referral to services and any other form of support needed by one of them. Many used personal agency to decide who to have sex with, to help ensure they felt secure and in control. We found that FSWs reported from early in their sex work career being able to understand the transactional nature of their relationship with clients and improve their condom negotiation skills as well as using PEP and PrEP. However, this was not always possible, especially when the client or a law enforcement agent physically or sexually abused them [38]. Sometimes sex workers -including those in Nairobi – campaign and advocate to make their occupation better and safer through collective action. These actions include championing the human rights of the group through collectives, attempts at decriminalization of sex work, building friendships among themselves, creating avenues to share issues/problems and ideas, starting economic empowerment projects such as saving groups and microfinancing schemes and lobbying for support from both local and international human right groups [27].

Community education has been shown to be a resource in promoting resilience among sex workers and can lead to improved confidence and self-efficacy [39]. Among sex workers, high self-esteem and self-efficacy are associated with healthy behaviours such as consistent condom use, better antiretroviral treatment adherence and better psychological well–being while the reverse is also true [40, 41]. Sexual health education gives women agency to practice safe sex and seek other promotive and preventive services such as PEP and PrEP [39, 42]. The empowerment and collective actions may result in group agency and social capital. Movements like ‘*nothing for us without us’* that advocate for meaningful involvement of sex workers in programs and interventions meant for them have facilitated the respect for the rights of female sex workers and improved their engagement in HIV programming and research [43]. The empowerment that results from support from HIV prevention and treatment programs is an important resource for nurturing resilience [44]. This was also reported in our study where the FSWs reported taking better care of themselves after interacting with SWOP.

Other factors that we found to play a role as resources for resilience were having known clients and being a parent. In Kibera, Kenya, sex workers reported that having regular clients can offer emotional and financial support [45]. On the other hand, this kind of safety net comes with challenges like the pressure to stop using condoms [46]. Hence, this needs to be explored further as it is not certain how many regular clients are enough or what would happen if they withdraw their support. We also found that parenthood and hope for a better future may have helped sex workers overcome suicidal thoughts, severe depression, and reduce alcohol and substance use. Our study findings add to this discourse by highlighting how parenthood may play a role in fostering resilience. Having children and in most instances, being the sole providers can motivate women to keep going. This intent to improve the lives of their children; speaks to findings from other studies [47].

When discussing plans for their future, many participants mentioned vague plans of starting a business when they have saved up sufficient capital; others hoped God would intervene and give them a man enabling them to leave sex work. Sex workers in our study, like many other sex workers in Kenya, have low education levels and lack training [48], locking them out of many alternative sources of livelihood and hindering them from exiting sex work. According to Hickle, there are four main types of exit from sex work, reactionary, gradual planning, natural progression, and *yo-yoing* [47]*.* The reactionary exit is usually in response to either something good or bad happening, for instance; marriage or facing a fatal experience among others. This type of exit, particularly when induced by a negative occurrence, is often temporary. This is because they do not get to plan on how they will make money outside sex work. Usually, the gradual exit is well planned over time and is a planned retirement. It is often in co-existence with an alternative source of income. It could be prompted by working conditions getting worse, competition getting stiff, or a simple decision to not want sex to sell forever. Sex workers exit over time by minimizing going outdoors (if they did that), and they also take in fewer clients. The natural progression type of exit is usually influenced by a strong desire to change a lifestyle. It could be because of circumstances like failed drug treatments, a chaotic lifestyle, old age, and mental and physical exhaustion. The yo-yo pattern is sometimes a consequence of a reactionary exit, for instance, after relieving psychological stress. Sometimes sex workers quit to do other jobs but come back to sex work when they cannot make as much money from other work [47]. Very few sex workers in our study had concrete plans on exit, which implies the eventual exiting from sex work will likely be reactionary, natural progression, or yo-yoing back and forth in and out of sex work.

### Study limitations

Participants were recruited from sex workers who were already accessing existing services and might not give a full picture of the challenges faced by all sex workers. However, the recruitment focused on active sex workers across all the Nairobi sub-counties hence their accounts of the lives of sex workers can be used as a representation of the experiences of other FSWs. Most of the participants were mothers and 25 years of age and above, as such the study cannot be generalised to the very young and those who do not have children. The information was self-reported and could be biased by selective memory and or misinformation bias (for example under-reporting of sensitive issues such as the experience of violence). Exploring resilience was not an objective in the study so we may not have captured all issues on the topic; however, resilience came out as a strong theme in the data even without it being a focus of the study.

## Conclusion

Going by the WHO definition of health which encompasses complete physical, mental and social wellbeing and not necessarily the absence of disease [49], a holistic approach will be needed in addressing the health needs of female sex workers. Access to mental health/ substance use/de-addiction services for sex workers as resources for resilience will go a long way in improving their quality of life. Outreach services that seek out women who are struggling and therefore not able to access services would help to find women when they are at a crisis point. Encouraging FSWs to come together and advocating together for their needs is a key resource from which resilience and forbearance can grow.

This would increase their negotiating power as a resilience asset, minimizing exposure and risk. Upstream prevention through strengthening of education systems and supporting girls to stay in school and complete their secondary and/or tertiary education would help them gain training and skills, providing them with options for income generation during their adult lives.

## Data Availability

The datasets used and/or analyzed during the current study are available from the corresponding author on reasonable request.

## Abbreviations

ACEs: Adverse Childhood Experiences
ARV: Anti-RetroViral
FSWs: Female Sex Workers
HIV: Human Immunodeficiency Virus
IPV: Intimate Partner Violence
MRC: Medical Research Council
NASCOP: National AIDS/STIs Control Program
PEP: Post-Exposure Prophylaxis
PHDA: Partners for Health and Development in Africa
PrEP: HIV Pre-exposure Prophylaxis
SWOP: Sex Workers Outreach Program
STI: Sexually transmitted infection
WHO: World Health Organization

## References

[1] Scorgie F, Chersich MF, Ntaganira I, Gerbase A, Lule F, Lo Y-R (2012). Socio-demographic characteristics and behavioral risk factors of female sex workers in sub-saharan africa: a systematic review. AIDS Behav.

[2] Cwikel JG, Lazer T, Press F, Lazer S (2006). Sexually transmissible infections among illegal female sex workers in Israel. Sex Health.

[3] Baral S, Beyrer C, Muessig K, Poteat T, Wirtz AL, Decker MR, Sherman SG, Kerrigan D (2012). Burden of HIV among female sex workers in low-income and middle-income countries: a systematic review and meta-analysis. Lancet Infect Dis.

[4] National AIDS and STI Control Programme (NASCOP). KENPHIA 2018 Report. Nairobi: NASCOP; 2020. https://www.health.go.ke/wp-content/uploads/2020/02/KENPHIA-2018-PREL-REP-2020-HR3-final.pdf.

[5] Platt L, Grenfell P, Meiksin R, Elmes J, Sherman SG, Sanders T, Mwangi P, Crago A-L (2018). Associations between sex work laws and sex workers’ health: A systematic review and meta-analysis of quantitative and qualitative studies. PLoS Med.

[6] Beyrer C, Pizer H (2007). Public Health and Human Rights: evidence-based approaches.

[7] Government of the Republic of Kenya: Penal Code. In., Laws of Kenya. Nairobi, Kenya: National Council for Law Reporting with the Authority of the Attorney-General,; 2012 [Rev.]. http://www.kenyalaw.org/lex/active.xql?actid=CAP.%2063.

[8] Mbote DK, Nyblade L, Kemunto C, Giger K, Kimani J, Mingkwan P, Njuguna S, Oga E, Kraemer JD (2020). Police discrimination, misconduct, and stigmatization of female sex workers in Kenya: associations with delayed and avoided health care utilization and lower consistent condom use. Health Hum Rights.

[9] Ma H, Loke AY (2019). A qualitative study into female sex workers’ experience of stigma in the health care setting in Hong Kong. Int J Equity Health.

[10] Kalemi G, Gkioka S, Tsapatsari P, Tzeferakos G, Kandri T, Psarra ML, Konstantopoulou F, Douzenis A (2017). Stigma and self-esteem: a case of HIV-positive sex-workers. Psychiatriki.

[11] Peitzmeier S, Mason K, Ceesay N, Diouf D, Drame F, Loum J, Baral S (2014). A cross-sectional evaluation of the prevalence and associations of HIV among female sex workers in the Gambia. Int J STD AIDS.

[12] Monroe J (2005). Women in street prostitution: the result of poverty and the brunt of inequity. J Poverty.

[13] Bronfenbrenner U, Vasta R (1992). Ecological systems theory. Six theories of child development: Revised formulations and current issues.

[14] Yates TM, Tyrell FA, Masten AS, Joseph S (2015). Resilience theory and the practice of positive psychology from individuals to societies. Positive Psychology in Practice.

[15] Fergus S, Zimmerman MA (2005). Adolescent resilience: a framework for understanding healthy development in the face of risk. Annu Rev Public Health.

[16] McCaghy CH, Hou C (1994). Family affiliation and prostitution in a cultural context: Career onsets of Taiwanese prostitutes. Arch Sex Behav.

[17] Ulibarri MD, Strathdee SA, Lozada R, Magis-Rodriguez C, Amaro H, O'Campo P, Patterson TL (2010). Intimate partner violence among female sex workers in two Mexico–U.S. Border cities: Partner characteristics and HIV risk behaviors as correlates of abuse. Psychol Trauma Theory Res Pract Policy.

[18] Baker LM, Dalla RL, Williamson C (2010). Exiting Prostitution: An Integrated Model. Violence Against Women.

[19] Bernstein E (2021). Temporarily Yours: intimacy, authenticity and the commerce of sex.

[20] Potter K, Martin J, Romans S (1999). Early developmental experiences of female sex workers: a comparative study. Aust N Z J Psychiatry.

[21] Parillo KM, Freeman RC, Collier K, Young P (2001). Association between early sexual abuse and adult HIV-risky sexual behaviors among community-recruited women☆. Child Abuse Negl.

[22] Swanston HY, Plunkett AM, O’Toole BI, Shrimpton S, Parkinson PN, Oates RK (2003). Nine years after child sexual abuse. Child Abuse Negl.

[23] Ferraro KF, Shippee TP, Schafer MH, Bengston VL, Gans D, Pulney NM, Silverstein M (2009). Cumulative inequality theory for research on aging and the life course. Handbook of Theories of Aging.

[24] Decker MR, Pearson E, Illangasekare SL, Clark E, Sherman SG (2013). Violence against women in sex work and HIV risk implications differ qualitatively by perpetrator. BMC Public Health.

[25] Bhattacharjee P, Campbell L, Thalinja R, Nair S, Doddamane M, Ramanaik S, Isac S, Beattie TS (2018). Understanding the Relationship Between Female Sex Workers and Their Intimate Partners: Lessons and Initial Findings From Participatory Research in North Karnataka, South India. Health Educ Behav.

[26] el Bassel N, Schilling RF, Irwin KL, Faruque S, Gilbert L, Von Bargen J, Serrano Y, Edlin BR (1997). Sex trading and psychological distress among women recruited from the streets of Harlem. Am J Public Health.

[27] Scorgie F, Vasey K, Harper E, Richter M, Nare P, Maseko S, Chersich MF (2013). Human rights abuses and collective resilience among sex workers in four African countries: a qualitative study. Glob Health.

[28] Fletcher D, Sarkar M (2013). Psychological Resilience. Eur Psychol.

[29] Rutter M (2007). Resilience, competence, and coping. Child Abuse Negl.

[30] Ledesma J (2014). Conceptual frameworks and research models on resilience in leadership. SAGE Open.

[31] National AIDS and STI Control Programme (NASCOP) (2019). Key population mapping and size estimation in selected counties in Kenya. Phase 1.

[32] Odek WO, Bhattacharjee P, Kimani J, Kimani G, Musyoki H, Githuka GN, Lorway R, Becker M, Isac S, Moses S (2014). Assessment of female sex workers' operational characteristics and programme exposure in Nairobi.

[33] Alliance ASW (2012). ‘Every sex workers has got a story to tell about violence’. Violence Against Sex Workers.

[34] Ngugi EN, Roth E, Mastin T, Nderitu MG, Yasmin S (2012). Female sex workers in Africa: Epidemiology overview, data gaps, ways forward. SAHARA-J.

[35] Widom CS, Kuhns JB (1996). Childhood victimization and subsequent risk for promiscuity, prostitution, and teenage pregnancy: a prospective study. Am J Public Health.

[36] Stoltz J-AM, Shannon K, Kerr T, Zhang R, Montaner JS, Wood E (2007). Associations between childhood maltreatment and sex work in a cohort of drug-using youth. Soc Sci Med.

[37] Beattie TS, Smilenova B, Krishnaratne S, Mazzuca A (2020). Mental health problems among female sex workers in low- and middle-income countries: A systematic review and meta-analysis. PLoS Med.

[38] Ross MW, Crisp BR, Månsson S-A, Hawkes S (2012). Occupational health and safety among commercial sex workers. Scand J Work Environ Health.

[39] Beattie TSH, Mohan HL, Bhattacharjee P, Chandrashekar S, Isac S, Wheeler T, Prakash R, Ramesh BM, Blanchard JF, Heise L (2014). Community mobilization and empowerment of female sex workers in Karnataka State, South India: associations with HIV and sexually transmitted infection risk. Am J Public Health.

[40] Yuen WW-Y, Wong WC-W, Tang CS-K, Holroyd E, Tiwari AF-Y, Fong DY-T, Chin WY (2013). Evaluating the effectiveness of personal resilience and enrichment programme (PREP) for HIV prevention among female sex workers: a randomised controlled trial. BMC Public Health.

[41] Wen J, Yeh T-P, Xie H, Yu X, Tang J, Chen Y. Resilience, self-esteem, self-efficacy, social support, depression and ART adherence among people living with HIV in Sichuan, China. AIDS Care 2021;33(11):1414–21. 10.1080/09540121.2020.1828800 33025792

[42] Blanchard AK, Mohan HL, Shahmanesh M, Prakash R, Isac S, Ramesh BM, Bhattacharjee P, Gurnani V, Moses S, Blanchard JF (2013). Community mobilization, empowerment and HIV prevention among female sex workers in south India. BMC Public Health.

[43] Anthony J, Hugar V, Munjattu JF, Pal A, Gyarappagari S, Ghosh S, Murugan S, Fahim S. Nothing for us without us: community led approaches towards successful implementation of HIV prevention programs: experiences from southern India. J Int AIDS Soc. 2012;15:264–5.

[44] Moore L, Chersich MF, Steen R, Reza-Paul S, Dhana A, Vuylsteke B, Lafort Y, Scorgie F (2014). Community empowerment and involvement of female sex workers in targeted sexual and reproductive health interventions in Africa: a systematic review. Glob Health.

[45] Benoit C, Roth E, Hallgrimsdottir H, Jansson M, Ngugi E, Sharpe K (2013). Benefits and constraints of intimate partnerships for HIV positive sex workers in Kibera, Kenya. Int J Equity Health.

[46] Aggleton P, O'Reilly K, Slutkin G, Davies P (1994). Risking everything? Risk behavior, behavior change, and AIDS. Science.

[47] Hickle KE (2017). Resiliency and women exiting sex trade industry work. J Soc Work.

[48] Odek WO, Githuka GN, Avery L, Njoroge PK, Kasonde L, Gorgens M, Kimani J, Gelmon L, Gakii G, Isac S (2014). Estimating the Size of the Female Sex Worker Population in Kenya to Inform HIV Prevention Programming. PLoS One.

[49] World Health Organisation Constitution [https://www.who.int/about/who-we-are/constitution].

